# Statistical modelling of biomarkers incorporating non-proportional effects for survival data

**DOI:** 10.1186/1745-6215-14-S1-P116

**Published:** 2013-11-29

**Authors:** Jacqueline Stephen, Gordon Murray, John Bartlett, David Cameron

**Affiliations:** 1 University of Edinburgh, Edinburgh, UK; 2 Ontario Institute for Cancer Research, Toronto, Canada; 3 Endocrine Cancer Group, Edinburgh University Cancer Research Centre, Edinburgh, UK; 4 Edinburgh Cancer Research Centre, Edinburgh, UK

## 

Personalised medicine is replacing the one-drug-fits-all approach with many prognostic models incorporating biomarkers available for risk stratifying patients with breast cancer, such as the Nottingham Prognostic Index and Adjuvant! Online and more recently multiparameter assays, OncotypeDx and Mammaprint.

Evidence of biomarkers having non-proportional effects have been emerging and therefore violating the assumption of proportional hazards when performing Cox regression. A classic example is the risk of recurrence after breast cancer depends on the duration of follow-up for estrogen receptor (ER) and progesterone receptor (PgR) expression status[1]. The gene signature MammaPrint has also been shown to have possible non-proportional effects with better prediction of patients at high risk of early relapse rather than those at risk of later disease progression[2,3].

A review of existing approaches for the analysis of non-proportional effects with respect to survival data found there to be a number of well-developed approaches for incorporating non-proportional effects but a lack of application of these approaches in practice. Two key approaches are the multivariable fractional polynomial time (MFPT) approach by Sauerbrei *et al.*[4] and flexible parametric models proposed by Royston & Parmer[5].

There is a need for more widespread use of flexible modelling to move away from standard analysis using a Cox model when the assumption of proportional hazards is violated. Fully determining the effects of markers in prognostic studies will help develop novel models for the selection of patients for appropriate treatments.
